# Factors Associated with Initiation of Opioid Use in a US Department of Veterans Affairs Pain Clinic: A Retrospective Study

**DOI:** 10.3390/brainsci15050491

**Published:** 2025-05-07

**Authors:** Anderson Chen, Erin Pleasants, Justine Lazatin, Naim Mekdessi, Christopher J. Miller, Diana Higgins, James Levitt

**Affiliations:** 1 Department of Geriatric Psychiatry, Massachusetts General Hospital, 55 Fruit Street, Boston, MA 02114, USA; achen49@mgh.harvard.edu; 2 Department of Psychiatry, Harvard Medical School, 25 Shattuck St., Boston, MA 02115, USA; 3 Center for Health Optimization and Implementation Research (CHOIR), VA Boston Healthcare System, 150 S Huntington Ave, Jamaica Plain, MA 02130, USA; 4 Harvard South Shore Psychiatry Residency Training Program, Brockton, 940 Belmont St., Brockton, MA 02301, USA; 5 Veteran Affairs Boston Healthcare System, 940 Belmont St., Brockton, MA 02301, USA; 6 Department of Psychiatry, Boston University School of Medicine, 750 Harrison Ave, Boston, MA 02118, USA; diana.higgins2@va.gov; 7 Veteran Affairs Durham Healthcare System, 508 Fulton St., Durham, NC 27705, USA

**Keywords:** chronic non-cancer pain, veterans, opioid use

## Abstract

Background: Guidelines suggest that, in chronic non-cancer pain (CNCP) management, non-opioid modalities should be prioritized, as there are negative consequences related to opioid use. There is a dearth of literature elucidating the risk factors for initiating opioid use amongst veterans with CNCP. Methods: Chart review on 193 patients with a new referral at a local US Department of Veterans Affairs (VA) pain clinic. Patients were required to have CNCP and not be taking opioid medication at the time of referral. The review started on 1 January 2014 and covered the year following. Data were analyzed via stepwise multiple logistic regression using Statistical Analysis System (SAS) software (Version 9.4; SAS institute Inc., Cary, NC, USA). Results: A total of 37 veterans (19%) received a new opioid prescription in the year following initial encounters at the pain clinic for CNCP. A history of substance use was associated with lower odds of receiving an opioid prescription. In contrast, being employed was associated with higher odds of receiving an opioid prescription. Conclusions: Amongst veterans treated for CNCP in a VA pain clinic, a history of substance use and a presence of vocation within the past year prior to presentation are variables associated with the initiation of opioids. Future studies to further elucidate the predictors of opioid prescriptions for CNCP are warranted.

## 1. Introduction

Opioid use contributes significantly to the morbidity and mortality of patients [1], including the veteran population [2]. One common reason for opioid initiation is the presence of pain [3]. However, negative consequences related to opioid use in CNCP patients include the development of an opioid addiction, the risk of diversion, and overdoses with serious medical sequelae [4]. Further, evidence suggests that opioid use may exacerbate pain control in the long term with the development of tolerance, hyperalgesia, and withdrawal [4].

Based on these factors, non-opioid treatments are now emphasized in the treatment of CNCP. Guidance from the CDC makes it clear that opioid management for chronic pain should not be common practice; on the contrary, non-opioid management should be considered first [5]. However, opioids continue to be prescribed for CNCP. Since the 1990s, opioid prescriptions have tripled [6]. The awareness and willingness of the US Department of Veterans Affairs (VA) to combat this issue is highlighted in its policies. Between 2012 and 2018, the VA reduced the amount of opioid prescribing by 64% through its Opioid Safety Initiative [7]. However, prescribing rates remain higher than those from the 1990s [8]. Recent research underscores the potential dangers of starting and stopping opioid treatment, suggesting that avoiding their use to treat CNCP in the first place may be safer than beginning—and then discontinuing—opioid prescriptions for CNCP [9].

Amongst those with opioid dependence, one of the primary indicators for opioid use is pain control [3]. Expert opinion from the 2022 CDC guidelines acknowledges that non-opioid treatment modalities should be maximized first, and opioid use in the context of chronic pain is not supported by meaningful evidence and is associated with harm [10]. Specifically, CNCP has a prevalence of about one in five US adults, with common causes such as back pain and arthritis [11]. Amongst those with CNCP, about 31% have an opioid prescription [11]. Traditionally, risk factors associated with opioid dependence for non-cancer pain include low socioeconomic status, mental health diagnoses, poor pain control, and a history of substance use [3]. Past studies examining the predictors of chronic opioid use, dependence, and higher dosage in veterans with CNCP outline risk factors including a history of substance use, psychiatric history, pain (back, joint, and neuropathic), female gender, an age range of 36–45, hepatitis C virus (HCV) diagnosis [12,13,14], protective factors of human immunodeficiency virus (HIV) positive status, and rural residency [15]. While other studies have demonstrated the impact of substance use and female gender on opioid initiation in CNCP in veterans, scarce evidence exists suggesting that age > 65 years impacts this. Conversely, no other studies have examined antibiotic use, inflammation markers (C-reactive protein (CRP) and the erythrocyte sedimentation rate (ESR)), vocation, and the use of non-opiate modalities have on risk factors for opioid initiation.

Considering that, in 2010, the lethality of chemical overdose was twice as high in the veteran population than in the general population [16], it is imperative to understand the risk factors for opioid initiation in context of CNCP in order to target efforts to reduce them. The current study investigates risk factors that are associated with the initiation of opioid prescription in the management of CNCP in the veteran population. Based on the current literature, we hypothesized that the strongest predictor among our chosen variables to examine is a history of substance use.

## 2. Materials and Methods

### 2.1. Ethics Statement

This study is a retrospective chart review. The local Institutional Review Board (IRB) deemed it exempt from IRB review, and it was then approved by the local Research and Development (R&D) Committee.

### 2.2. Data Source

The VA Corporate Data Warehouse (CDW) was the repository for the data entered into the Computerized Patient Record System (CPRS), the VA electronic medical record. We used the CDW to identify patients with new referrals to the local VA pain clinic meeting the inclusion criteria outlined below. A manual review of electronic health records (EHRs) was conducted to perform data extraction using a standardized chart review tool that was developed prior to conducting chart reviews, including 1 additional independent reviewer who overlapped on selected charts to ensure reliability.

### 2.3. Cohort

Our cohort included all new referrals to a local VA pain clinic since 1 January 2014 who were (a) referred for CNCP, (b) not on an opioid medication, and (c) had been opioid free for the year prior to the initial clinical encounter in the pain clinic. The last initial pain clinic encounter date for any subject included in the study is in late 2020, and, thus, the last follow-up period for any subject in the study ended in late 2021. We used the stop code 420 in the pain clinic to identify patients with CNCP, which refers to chronic pain not caused by cancer. We chart reviewed all qualifying patients. The initial clinic encounter that identified our opioid-free patients was the “index date”; then, we identified covariates (described below) and followed for one year forward from the index date to determine whether patients remained opioid free or were prescribed an opioid medication for any duration and from any prescriber. The study end points were either (a) when a patient was prescribed an opioid or (b) by end of the 1-year follow-up period from the index date.

### 2.4. Risk Factors Analyzed

We analyzed 12 risk factors, including 4 demographic, 3 clinical, and 5 medical risk factors. Covariates were measured in the one year prior to the index date.

Demographic risk factors included age, gender, marital status, and vocation. We categorized age as young (20–40), middle-aged (41–65), and 65 yrs and above at the index date. Sex at birth was treated as binary (female/male). Marital status was defined as single/married/divorced as last documented at or before the index date. Vocation was determined via chart review by having had at least 1 job within 1 year prior to the index date.

Clinical risk factors analyzed included the presence of post-traumatic stress disorder (PTSD), body mass index (BMI), and a history of substance use. The presence of PTSD via chart review as defined as 2 outpatient or 1 inpatient contact for PTSD in the 12 months prior to the index date, with binary outcomes of yes/no, as has been used in prior research using VA administrative data [17]. BMI, as defined by “weight in kilograms divided by the square of height in meters [18]”, was determined based on the weight and height last documented at or before the index date. A history of substance use was determined via chart review as 2 outpatient or 1 inpatient contact for substance use in the 12 months prior to the index date.

The medical risk factors analyzed included pain localization, pain severity, elevated CRP or ESR, and antibiotic use. The inclusion of ESR and CRP was used to reflect that common causes of CNCP include rheumatoid arthritis and fibromyalgia [3]; the inclusion of antibiotic use was used to reflect recent studies suggesting the microbiome’s impact on pain regulation [19]. Pain localization was determined as one pain source (local) versus more than one pain source (non-localized) by the index date. Pain severity was determined by averaged pain intensity numeric rating scale scores on chart review 1 year prior to the index date. CRP and ESR elevation were determined by the presence of one elevated reading of CRP or ESR in the 1 year prior to the index date. Antibiotic use was determined as a binary (Y/N), as indicated by any antibiotic prescription in the VA 1 year prior to the index date. The presence or absence of non-opioid treatment modalities included Cognitive Behavioral Therapy (CBT), Physical Therapy (PT), Occupational Therapy (OT), acupuncture, and invasive procedures.

### 2.5. Statistical Analysis

The data were analyzed using SAS software (Version 9.4; SAS institute Inc., Cary, NC, USA), which provided the results of the stepwise backwards selection method to determine the variable importance. We then placed the recommended variables from the procedure into a multiple logistic regression model. We used a backwards selection stepwise approach to determine which covariates should be retained in the multivariate model, with an alpha of 0.3 to enter the regression model and an alpha of 0.35 to stay within the regression model [20]. These criteria were selected to support an inclusive modeling strategy, allowing for the identification of potentially important covariates, mainly due to sample size limitations. The alpha levels were consistent with the recommendations for exploratory analyses and an emphasis on predictive accuracy [21]. The risk factors identified above were the independent variables, while opioid prescription was the dependent variable. Based on these criteria, the variables of PTSD, BMI, marital status, pain localization, pain severity, and ESR and CRP status were not retained in the statistical model, as theses variables did not meet the criteria to be included in the regression model. A multiple logistic regression model was used to assess the association between these above predictor variables (gender, antibiotic use, vocation, non-opioid treatment, and a history of substance use) and the likelihood of opioid prescription initiation for CNCP as a binary outcome variable. The logistic regression model estimated odds ratios (ORs) and 95% confidence intervals (CIs) for each predictor. Statistical significance was evaluated at an alpha level of 0.05. The overall model significance was determined by the likelihood ratio chi-square test. All of the analyses were conducted using SAS software (Version 9.4; SAS Institute Inc., Cary, NC, USA).

## 3. Results

### 3.1. Patient Sample

We identified 193 patients meeting the inclusion criteria, of whom 37 (19.2%) were prescribed an opioid medication during the follow-up period by different clinicians and 156 (80.8%) were not. Overwhelmingly, the sample size population was male (92%), with only 31% of the sample size categorized as an older adult (age 65+).

Amongst those with an opioid prescription, 46% were above the age of 65, 86% were males, 38% had antibiotic use within past year, 54% had a history of substance use, 43% had localized pain, 38% were married, 35% had a job within the past year, and 38% had the presence of PTSD. Amongst those without an opioid prescription, 28% were above the age of 65, 94% were males, 22% had antibiotic use within the past year, 31% had a history of substance use, 48% had localized pain, 59% were married, 53% had a job within the past year, and 38% had the presence of PTSD. Table S1 contains the complete descriptive statistics for the sample.

### 3.2. Multiple Logistic Regression

The statistically significant variables at an alpha level of 0.35 included a history of substance use, vocation, gender, non-opioid treatments, and antibiotic use. A multiple logistic regression analysis was then performed to evaluate the association between the above predictors and the initiation of opioid prescriptions. The overall model was statistically significant, indicating a strong baseline likelihood of opioid prescription initiation in the absence of other predictors. Gender (*estimate = 1.1027*; *χ^2^ = 2.9569*; *p = 0.0855*) approached statistical significance, suggesting that males may be more likely to initiate opioid prescriptions compared to females, though this effect did not reach the suggested level of a 0.05 alpha significance. Antibiotic use (*estimate =* −*0.5174*; *χ^2^ = 1.4944*; *p = 0.2215*) and non-opioid treatment (*estimate = −0.6882*; *χ^2^ = 1.5047*; *p = 0.2199*) were not significant predictors of opioid initiation, indicating that these factors did not substantially influence whether patients were prescribed opioids in this cohort. However, a history of substance use was a significant predictor (*estimate = −1.0449*; *χ^2^ = 7.0322*; *p = 0.0080*). Individuals with a history of substance use were significantly less likely to initiate opioid prescriptions, suggesting that clinicians may have been cautious about prescribing opioids to this group. Vocation (*estimate = 0.9419*; *χ^2^ = 5.1196*; *p = 0.0237*) was also found to be a significant predictor of opioid initiation, but, in contrast, it was a positive predictor, suggesting that employment history was also a factor in prescribing patterns for chronic pain management. For a summary, see Table S2.

## 4. Discussion

In our retrospective chart review study of veterans presenting to a pain clinic with CNCP, a history of substance use was associated with a lower risk of a veteran being initiated on an opioid. This is indeed a hypothesis–result mismatch, as we originally predicted a history of substance use to be the strongest predictor of opioid initiation. The literature traditionally recognizes a history of substance use as a predictor of receiving an opioid medication [3,22] or the subsequent development of opioid dependence [14]. This discrepancy could be due to changes in attitude towards opioid prescription amongst clinicians, or our novel approach of looking at opioid initiation as opposed to the general risk factors of receiving an opioid, which would include opioid initiation, maintenance, and re-initiation within 1 year of the last opioid prescription. This finding is reassuring, in that, intuitively, it is reasonable to surmise that the use opioids in patients who have a substance use disorder increases the risk of the misuse or overuse of pain medication. This clinical caution related to a history of substance use and opioid initiation would benefit from further studies to create empirical support. Certainly, alternative explanations should be considered. Our data are derived from a pain clinic located in the northeastern United States; as such, our results may have been influenced by single-site cultural bias or prescriber bias. As noted in our limitations, we also do not know how many prescribers were providing opioids during our study period.

Our findings also suggest that having at least one job within the year prior to presentation to a pain clinic is associated with a higher chance of opioid initiation. This is a novel finding, though more studies need to be performed to corroborate this. This finding does signal an important consideration for clinicians when weighing treatment options for CNCP. The above association is of potential interest, as it is possible that clinicians may give greater benefit of the doubt to prescribing an opioid for an employed patient when such a patient may not require such a treatment any more than an unemployed patient. Lastly, we note that female sex assigned at birth was associated with a slightly lower risk of initiating an opioid medication, but this finding was not statistically significant. Given that less than 10% of our sample was female, we were likely underpowered to detect significant effects based on this variable.

These findings should be considered in the context of previous literature examining opioid use among veterans. A prospective cohort study within the VA found that long-term opioid therapy (LTOT) is associated with other risk factors, such as a younger age, obesity, and less medical comorbidities, as well as being male [23]. There are discrepancies to the LTOT risks identified in this study compared to the risks that we identified for opioid initiation. It would thus be informative for future studies to investigate why such differences exist. In Edlund and colleagues’ study of 15,160 veterans who use opioids chronically, those who developed opioid abuse or dependence most likely had a concomitant substance use history (OR: 2.34; *p* < 0.01), as well as having a psychiatric history (OR = 1.46; *p* = 0.005) [14]. In Morasco and colleagues’ study of 19,677 veterans, those being prescribed a high dose of an opioid medication—defined by greater than 180 mg/day of morphine equivalent—were more likely to have experienced “neuropathy, low back pain, and nicotine dependence diagnoses [13]”. Other past studies in the veteran population have focused on risk factors related to opioid overdose risk [24]. Although the other two studies in the veteran population had large sample sizes, while ours did not, we believe that our study still adds to the literature. In addition to our extended follow-up period of 1 year, our study is different from past studies, in that we examined the risk of initial opioid prescription in the context of having chronic non-cancer pain. This is an important gateway for a high-risk population to be exposed to opioids, where many may subsequently develop misuse and abuse. Past studies have shown that 77% of veterans who are prescribed an opioid are also prescribed “psychotropic medications”, including anxiolytics [25]. As such, this is also a gateway for the start of a potentially troublesome pattern of polypharmacy prescribing. To summarize, our focus on opioid initiation in the context of the patient/provider relationship—as opposed to receipt of the opioid itself or the subsequent development of opioid dependence—is a novel aspect of our study. The risk factors identified here are patient characteristics that may impact a prescriber’s choice to initiate, or not initiate, an opioid prescription for a patient who has been opioid free for at least 1 year. Further studies would be needed to corroborate our findings.

## 5. Limitations

These findings should be considered in the context of several limitations. Our study was relatively small and focused on one clinical site, and we acknowledge that our sample size of 197 veterans, of which only 37 were prescribed an opioid, may not have been sufficient to analyze the 12 risk factors. Future studies will benefit from a larger sample size from different clinical sites and further information, including the types of opioids prescribed, as well as the number of doctors prescribing opioids in the pain clinic and their prescribing habits, during the study period. The retrospective study allowed us to examine risk factors prior to the intervention, specifically a clinical encounter in a specialty pain clinic. However, it also limited the examination of the presence of PTSD or substance use to the year prior to the initial pain clinic visit. Along this vein, chronic conditions or other historical factors may not be fully captured. The findings may not generalize to those with prior diagnoses of these conditions or non-veteran populations. Similarly, our one-year follow-up period meant that we could not speak to the potential longer-term impacts of our covariates on opioid prescribing patterns. Given that only 37 veterans were prescribed an opioid from our sample size of 193, we may have had limited power to detect significant effects. It is possible that a remote history of opioid therapy or opiate use disorder—undocumented in the chart—could have impacted our results. Unmeasured confounding is always a risk for retrospective studies. As with retrospective chart reviews, the veracity of the diagnosis for chronic pain has the limitations of administrative data, via CPRS in this case. Many chart review studies have been conducted in the VA, and past studies suggest that CPRS diagnoses can be used to derive meaningful research findings [26,27]. It is important to note that we did not investigate the duration of the therapy used, and future studies would benefit from stratifying whether a varied degree of engagement of therapy would have led to a different outcome. Also, CNCP is a heterogenous term that includes different modalities of pain, such as musculoskeletal and neuropathic pain. Future studies would benefit from investigating specific pain modalities or, perhaps, by using specific DSM-5 or ICD-11 codes. Furthermore, our sample was drawn beginning in 2014, and, of course, attitudes and guidelines around opioid prescribing have changed since then. We note, however, that the VA’s Opioid Safety Initiative—which aimed to curb high-risk opioid prescribing—was first launched in 2013, before our data collection began [28]. Having said that, new guidelines were released in 2022 [10]. We believe a future replication study of patients similarly in a pain clinic, such as that of our patients, at a later time period, after the new guidelines went into effect, would be a highly interesting and informative additional study to perform. Without doubt, we acknowledge that a limitation of our study is that evolving policies regarding opioid prescription might have influenced the prescribing patterns. Lastly, while this study only examined prescriptions started in a pain clinic, most opioid prescriptions in the veteran population are written by primary care [29]. Our findings are most likely to apply to veterans seeking care in pain clinics, and we caution against generalizing to veterans seeking care in other clinical settings.

## 6. Conclusions

Our data add to the very limited literature examining risk factors amongst veterans being prescribed an opioid in context of chronic non-cancer pain (CNCP). In a retrospective chart review, where a minority of patients were prescribed an opioid for CNCP, we identified a history of substance use as a statistically significant risk factor for predicting lower odds of opioid initiation within 1 year of a follow-up period after seeing a pain specialist. Conversely, having had a job within past year prior to seeing a pain specialist was associated with a higher likelihood of being prescribed an opioid during a 1-year follow-up period. The clinical relevance of this should be explored further in future studies, including how vocation is beneficial, such as its impact on pain severity or functional status, and its potential to provide a useful distraction from focusing on pain symptoms.

We caution against extrapolating our data on how one might consider risk factors for the difficulty of tapering off an opioid prescription for CNCP for those already receiving an opioid prescription, as this is not our study population. Rather, we believe the risk factors that we have identified here may help clinicians to consider preventive measures prior to the initiation of opioids in veterans with CNCP. In the context of concerted efforts to minimize opioid prescriptions among populations vulnerable to misuse and addiction, patients with a history of a documented diagnosis of substance use in our sample are now less likely than their peers to be prescribed an opioid after controlling for relevant covariates. A narrative review suggests that the incidence of opioid use disorder and overdose increases after surgery [30]; this would be a worthy factor to control for in future studies. Lastly, we also note that future work might include the assessment of prescriber characteristics as well as patient characteristics for the risk factors associated with the initiation of opioids in patients with CNCP.

## Data Availability

The data presented in this study are available on request from the corresponding author due to privacy restrictions.

## Supplementary Materials

The following supporting information can be downloaded at: https://www.mdpi.com/article/10.3390/brainsci15050491/s1, Table S1: Descriptive statistics for the sample; Table S2: Analysis of maximum likelihood estimates.
