# Supplementary material for: Factors Associated with Initiation of Opioid Use in a US Department of Veterans Affairs Pain Clinic: A Retrospective Study
Source: Brain Sci. 2025 May 7;15(5):491. doi: 10.3390/brainsci15050491 (PMC12109717; doi:10.3390/brainsci15050491)
Supplement: Supplementary file 1 [file brainsci-15-00491-s001.zip › brainsci-3588055-supplementary.pdf]

Supplementary Materials:

Table S1. Descriptive Statistics for the Sample

	Total Sample (N=193) (%)	With Opioid Prescription (N=37) (%)	Without Opioid Prescription (N=156) (%)
**Age 65+**	*61 (31.61)*	*17 (45.95)*	*44 (28.21)*
**Age 41-65**	*89 (46.11)*	*16 (43.24)*	*73 (46.79)*
**Age 20-40**	*43 (22.28)*	*4 (10.81)*	*39 (25)*
**Male**	*178 (92.23)*	*32 (86.49)*	*146 (93.59)*
**Female**	*15 (7.77)*	*5 (13.51)*	*10 (6.41)*
**Prescribed Opioid**	*37 (19.17)*	*37 (100.00)*	*0 (0.00)*
**Antibiotic use**	*48 (24.87)*	*14 (37.84)*	*34 (21.79)*
**Substance Use**	*69 (35.75)*	*20 (54.05)*	*49 (31.41)*
**Localized Pain**	*91 (47.15)*	*16 (43.24)*	*75 (48.08)*
**Married**	*106 (54.92)*	*14 (37.84)*	*92 (58.97)*
**With job**	*96 (47.63)*	*13 (35.14)*	*83 (53.21)*
**Elevated CRP**	*7 (3.62)*	*1 (2.70)*	*6 (3.85)*
**Elevated ESR**	*3 (1.55)*	*0 (0.00)*	*3 (1.92)*
**Presence of PTSD**	*73 (37.82)*	*14 (37.84)*	*59 (37.82)*
**Had CBT**	*73 (37.82)*	*17 (45.95)*	*56 (35.90)*
**Had PT/OT**	*69 (35.75)*	*14 (37.84)*	*55 (35.26)*
**Had Acupuncture**	*30 (15.54)*	*3 (8.11)*	*27 (17.31)*
**Had Invasive Procedure**	*97 (50.25)*	*16 (43.24)*	*81 (51.92)*
**BMI, Average**	*29.98*	*30.65*	*29.82*
**Pain Severity, (Average score from 1-10)**	*3.85*	*4.01*	*3.94*

CRP= C-reactive protein, ESR= Erythrocyte Sedimentation Rate, PTSD= Post-traumatic stress disorder, CBT= Cognitive Behavioral Therapy, PT= Physical therapy, OT= Occupationa therapy, BMI= Body Mass Index.

Table S2. Analysis of maximum likelhihood estimates

Parameter	DF	Estimate	OR (CL)	Standard Error	Wald Chi-Square	Pr > ChiSq

**Intercept**	1	*2.3260*	*.*		*0.6191*	*14.1169*	*0.0002*
**Gender**	1	*-1.1027*	*0.332 (0.094, 1.167)*		*0.6413*	*2.9569*	*0.0855*
**Antibiotics**	1	*-0.5174*	*0.596 (0.260, 1.366)*		*0.4232*	*1.4944*	*0.2215*
**Substance Use**	1	*-1.0449*	*0.352 (0.162, 0.761)*		*0.3940*	*7.0322*	*0.0080*
**Vocation**	1	*0.9419*	*2.565 (1.134, 5.799)*		*0.4163*	*5.1196*	*0.0237*
**Non-Opioid Treatment**	1	*-0.6882*	*0.502 (0.167, 1.509)*		*0.5610*	*1.5047*	*0.2199*
